# Local Variability Mediates Vulnerability of Trout Populations to Land Use and Climate Change

**DOI:** 10.1371/journal.pone.0135334

**Published:** 2015-08-21

**Authors:** Brooke E. Penaluna, Jason B. Dunham, Steve F. Railsback, Ivan Arismendi, Sherri L. Johnson, Robert E. Bilby, Mohammad Safeeq, Arne E. Skaugset

**Affiliations:** 1 Pacific Northwest Research Station, United States Forest Service, 3200 SW Jefferson Way, Corvallis, OR, 97331, United States of America; 2 Department of Fisheries and Wildlife, Oregon State University, 104 Nash Hall, Corvallis, OR, 97331, United States of America; 3 U.S. Geological Survey, Forest Rangeland Ecosystem Science Center, Corvallis Research Group, 3200 SW Jefferson Way, Corvallis, OR, 97331, United States of America; 4 Lang Railsback & Associates, 250 California Avenue, Arcata, CA, 95521, United States of America; 5 Weyerhaeuser Company, Post Office Box 9777-WTC 1A5, Federal Way, WA, 98063, United States of America; 6 Sierra Nevada Research Institute, University of California Merced, Merced, CA, 95343, United States of America; 7 Department of Forest Engineering, Oregon State University, Corvallis, OR, 97331, United States of America; Northwest Fisheries Science Center, NOAA Fisheries, UNITED STATES

## Abstract

Land use and climate change occur simultaneously around the globe. Fully understanding their separate and combined effects requires a mechanistic understanding at the local scale where their effects are ultimately realized. Here we applied an individual-based model of fish population dynamics to evaluate the role of local stream variability in modifying responses of Coastal Cutthroat Trout (*Oncorhynchus clarkii clarkii*) to scenarios simulating identical changes in temperature and stream flows linked to forest harvest, climate change, and their combined effects over six decades. We parameterized the model for four neighboring streams located in a forested headwater catchment in northwestern Oregon, USA with multi-year, daily measurements of stream temperature, flow, and turbidity (2007–2011), and field measurements of both instream habitat structure and three years of annual trout population estimates. Model simulations revealed that variability in habitat conditions among streams (depth, available habitat) mediated the effects of forest harvest and climate change. Net effects for most simulated trout responses were different from or less than the sum of their separate scenarios. In some cases, forest harvest countered the effects of climate change through increased summer flow. Climate change most strongly influenced trout (earlier fry emergence, reductions in biomass of older trout, increased biomass of young-of-year), but these changes did not consistently translate into reductions in biomass over time. Forest harvest, in contrast, produced fewer and less consistent responses in trout. Earlier fry emergence driven by climate change was the most consistent simulated response, whereas survival, growth, and biomass were inconsistent. Overall our findings indicate a host of local processes can strongly influence how populations respond to broad scale effects of land use and climate change.

## Introduction

Although most studies that have considered the combined effects of land use and climate change have focused on range shifts at the edges of species distributions [1–3], it is likely that many responses to both land use and climate change occur well within these edges, with changes in population demography that precede detectable changes in species distributions [4]. Although several studies have addressed how population demography is affected by climate change [4, 5–8], land use [9–11], or their combined effects [12–13], few studies have focused on disentangling the underlying mechanisms explaining these changes [5, 8]. Within the range of a species, it is reasonable to expect increased variation in local environmental conditions initially, with more homogeneous and coherent changes manifested as the effects of land use and climate change intensify [14]. Accordingly, local variability in environmental conditions may play a key role for understanding emerging responses to land use and climate change, but it has yet to be fully explored [15–18]. Local environmental conditions create unique microclimates or “idiosyncrasies of place” where individuals and populations of species experience change [17]. In the context of land use and climate change, an improved understanding of such local variability can provide critical insights into processes behind observed changes in species distribution and abundance [19].

**Fig 1:**
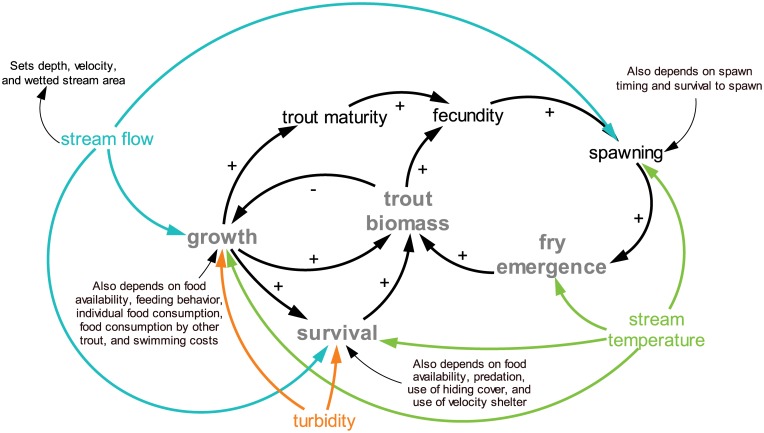
Representation of Key Processes in inSTREAM. We highlight how the daily time series inputs of stream temperature, flow, and turbidity drive individual growth and survival and hence population dynamics including responses of fry emergence and biomass. A more detailed explanation of inSTREAM can be found in [20–21].

Here we consider local variability in the vulnerability of stream-living trout to changes in temperature and flow linked to forest harvest and climate change. We used an individual-based model of Coastal Cutthroat Trout (*Oncorhynchus clarkii clarkii)* population dynamics [20–21] to model the separate and combined effects of stream temperature and flow under forest harvest and climate change scenarios on trout responses of biomass, survival, growth, and timing of fry emergence in four neighboring streams located within the same headwater catchment (Fig 1). We parameterized the model with multi-year, daily measurements of stream temperature, flow, and turbidity (2007–2011), and field measurements of both instream habitat structure and three years of annual trout population estimates. The forest harvest scenario encompassed two harvests leading to increased stream temperature in summer [22], increased summer flows, and increased magnitudes of storm events [23–24] for 1–2 decades following each simulated harvest [23,25]. The climate change scenario involved increasing stream temperature year-round [26] and decreasing flow during fall and winter [27]. The combined scenario captured the simultaneous effects of forest harvest and climate change, allowing us to evaluate the interactive effects of these changes on the dynamics of local populations.

## Methods

### Study sites

Our modeled streams are based on actual headwater streams in the Trask Watershed in northwestern Oregon, USA (S1 Fig); this location is part of a regional effort to understand the effects of forest harvest and climate change on fish (http://watershedsresearch.org/trask/). We simulated Gus Creek, Upper Mainstem Trask, Pothole Creek, and Rock Creek. Precipitation in the Trask Watershed study area occurs primarily during winter and varies by elevation, with rain at lower elevations and a mix of snow and rain at higher elevations. These climatic conditions, combined with steep terrain and shallow soils, lead to flashy winter flows linked to winter storms, followed by declining flows in spring and an extended low flow period in late summer [27, 28]. Our study period captured three years that were marginally higher in mean annual precipitation (2758 mm · y^-1^, 2834 mm · y^-1^, 2783 mm · y^-1^), one year that was lower (2255mm · y^-1^), and one representative year (2626 mm · y^-1^) when compared to the long-term annual mean (2500 mm ·y^-1^) for the Trask River watershed [29]. Daily mean stream temperatures were moderate year-round ranging from 6–12°C. The fish communities of these streams were dominated by resident Coastal Cutthroat Trout *Oncorhynchus clarkii clarkii*, with sculpins *Cottus* spp., juvenile Steelhead *O*. *mykiss*, and juvenile Coho Salmon *O*. *kisutch* at some sites. Movement of fish among study streams consists of a very small rate of exchange (<1%, J. Dunham, unpublished data), which is likely not important demographically [30]. In these coastal streams, resident Coastal Cutthroat Trout spawn in the spring generally in April and May with fry commonly emerging from April to August [31]. Although trout grow all year, most growth for age 1+ trout occurs in winter and spring [32].

Streams varied in their local features (S1 Table; [33]). Average summer flows were similar among all streams, but flows in Rock Creek were more variable in summer. Although Rock Creek was the largest stream in watershed area, it had similar availability of habitat in summer relative to Gus Creek, which is half its watershed area. Gus Creek has the most variable flow in winter, and at times it exceeded flows in Rock Creek. Upper Mainstem was the coldest stream year-round, and it was the smallest in both watershed area and wetted summer area. Pothole Creek had the highest and most variable turbidity in winter and the lowest in summer.

### The trout model

The individual-based trout model we used was version 5.0 of inSTREAM, downloaded August 30, 2014 (Fig 1). This model has been fully described, and is publically available ([20, 21]; http://www.humboldt.edu/ecomodel). Unless noted here, default parameter values for Cutthroat Trout and small streams were extracted from the literature [20, 33] and details of model calibration are in [3]. InSTREAM has shown realistic trout responses for individuals [34] and populations [35–37]. Details of model calibration can be found in Penaluna et al. [33]. Although we calibrated inSTREAM to each stream using three years of trout population estimates from each field site, values of model calibrations were the same at three of the four streams, except for Upper Mainstem (S1 Table). Of the parameters considered in a sensitivity analysis (baseflow, benthic and drift food, summer temperature, hiding cover, piscivory risk, redd scour, spawning gravel, velocity shelter, winter temperature), biomass of adult trout was consistently sensitive to baseflow, with higher baseflows resulting in greater biomass of adult trout for these streams [33]. Although there are multiple stochastic elements in inSTREAM, it is not a highly stochastic model. Mortality is the main process represented stochastically. Other stochasticity occurs in initialization of model run (various aspects), assignment of length when trout emerge from eggs, whether a female that is ready to spawn does on an actual day, and mortality of eggs [20, 21]. Hence, differences in replicate model runs represent natural variability and uncertainty in stochastic processes.

InSTREAM captures many complexities of real streams. Within the inSTREAM environment, each study stream was represented as a reach (here, 210–250 m in length) made up of rectangular cells (here, 31–35 cells per reach). Reach-scale variables included flow, stream temperature, and turbidity, which varied daily. Each cell represented a unit of microhabitat from one to several square meters in area that had a specific depth, velocity, area of velocity shelter for drift-feeding, area of spawning gravel, and distance to hiding refuge. Stream depth and velocity varied daily with flow. Trout were represented as freely feeding individuals following emergence from gravel. Each trout was attributed with a length, weight, condition, and sex, and was assigned to a cell within which it feeds. Because trout were modeled as individuals, population dynamics emerged from the behaviors and fates of individual fish. The complete trout life cycle was represented in the model (redds, egg, fry, juvenile, adult). Redds were represented by both the number of eggs they contain and egg developmental status.

InSTREAM operated at a daily time step following a set schedule of actions (in order: spawning, habitat selection, growth, survival, and egg development). At the start of each day, daily flow, temperature, and turbidity were taken from input files for each scenario, which are based on actual field observations. Depth and velocity for each habitat cell were updated based on the flow input. Hence, these daily updates allowed for changing environmental conditions corresponding to each scenario (see scenarios and model input sections for more details).

Female trout spawn once per year if conditions, including date, flow and temperature thresholds, and female size, were met. When conditions for spawning were met, female trout moved to a cell with spawning gravel and created a redd. The number of eggs deposited increased geometrically as a function of spawner fork length. Each breeding adult (female and male) incurs a weight loss of 20% to represent energy loss due to reproduction. The model follows egg survival, development, and emergence. The developmental stage of eggs within a redd was a function of temperature degree days. Each day, the model updates developmental stage, determining how many eggs die due to processes such as temperature stress and disease, scouring, or desiccation. The timing of spawning was important because flow and temperature fluctuations, both high and low, influenced egg mortality. When surviving eggs within a redd were fully developed, they were converted into new trout, analogous to emergence and first feeding in nature.

All trout selected a cell and feeding activity to maximize short-term (90 day) fitness, which was a function of the growth and survival probability offered by each potential cell [35]. Habitat selection was executed in order of fish size, with the largest fish selecting cells first, representing a length-based hierarchy. The version of inSTREAM we used assumes that trout fed during daylight hours and competed for the food available in each cell [20]. Both metabolic and activity costs are subtracted from growth (net energy intake) and growth emerges as a function of trout size and habitat conditions (depth, and velocity). However, trout can also lose energy during spawning season (if they mature and if they spawn). Food intake was limited by food availability and the trout’s ability to capture food. Food availability depends on how much food was in each cell and how much was consumed by competing trout. The ability to capture food depends on trout size (with increasing length because larger trout see and swim better) and habitat conditions. There are two modes of feeding for trout, drift-feeding or search feeding. Food intake was represented with a drift-feeding approach where trout capture invertebrates as they are carried within range by the current. Search feeding is active searching for food. It is the primary feeding behavior for young-of-year and consists of 10–20% of food supply for adults [36]. The survival probability offered by a cell was determined by functions representing mortality due to predation by terrestrial animals, trout predation, disease and starvation, and high temperature. These functions depended on individual length and weight and on habitat variables. Hence, trout grew and survived according to growth rate and survival probabilities of the selected cell.

### Scenarios

We designed this study to mimic changes in stream temperature and flows associated with separate and combined effects of forest harvest and climate change. In addition to these changes, we modeled a baseline scenario that mimicked the natural variability in temperature and flow observed from 2007–2011 at each stream. We generated 63 years of results for each scenario (duration explained in detail in section on model input). To account for two forest harvests on a 40-year rotation, we applied changes to stream flow and temperature due to forest harvest in years 1 and 41 for all of our analyses. To understand the influence of each environmental regime, we also compared the separate and combined effects of stream temperature and flow for each scenario.

To model the effects of forest harvest, we comprehensively assessed information from recent forest harvest studies in the region. We used a conservative scenario of changes in magnitude and timing of stream temperature and flow. Accordingly, our forest harvest scenario represented a composite of current practices that include riparian buffers and a 40-year rotation. More generally, the literature suggested increased summer temperatures, increased summer flows, and increased flow from winter storm events for 1–2 decades following each harvest. Hence, for each cutting, we elevated stream temperatures for 15 years, with the first five years having daily mean stream temperature increased by 0.37°C [22] uniformly, followed by a gradual, linear decrease in daily mean stream temperature by 0.037°C each year for 10 years until recovery of baseline conditions [25]. Therefore, baseline temperature values were restored in year 16 and again in year 57 because that is when the forest canopy would be expected to be restored, providing shade to the stream. Responses of flow regimes to forest harvest are variable, but in the Coast Range of Oregon, alterations to flow regimes can last for 20 years with changes to storm events and seasonal low flows [23]. We increased daily water yield by 20% on the days when a storm event occurred that generated flow > 2 standard deviations above the annual mean, because although water yield increases from forest harvest are highly variable, they generally increase by approximately 20% [23–24]. We increased summer (July-September) daily mean flows by 45% above baseline for five years [23, 38], then decreased summer mean flows by 2.25% per year for 15 years until baseline was reached in year 20 post-harvest and again in year 60, because as the forest regrows, more water is used by trees.

We generated the climate change scenario by increasing year-round stream temperatures and decreasing fall and winter flows. We increased stream temperatures by 0.06°C per year, a rate based on stream temperature trends occurring in the Pacific Northwest over the past 50 years [26], reaching 3.78°C higher than baseline at the end of the study period. This final increase in stream temperature is within the range of predicted warming for the Pacific Northwest [39]. We decreased mean fall flows by 0.25% (October-December) and mean winter flows by 0.49% (January-March) each year [27], resulting in a gradual decrease over time that reached 15.75% below baseline for fall flows and 30.87% below baseline for winter flows at the end of the simulation. Streams of coastal Oregon generally have hydrographs dominated by rainfall, not snowmelt and, hence, key changes related to climate change are occurring during peak winter months due to decreasing rain and not during spring and summer snowmelt [27].

For the combined scenario, we first made the changes from forest harvest and then added alterations from climate change.

### Model Input

We parameterized the model for each stream with field measurements of habitat features recorded during seasonal low flow (August-September) in 2009. Measurements of habitat were completed and used to delineate cells. For each cell, we measured availability of velocity shelter and spawning gravel as well as distance to hiding cover. Depth, velocity, and water surface elevation of each cell were measured over a range of low, medium, and high flows from 2009–2010. We computed daily mean (arithmetic) of flow, stream temperature, and turbidity from field measurements recorded every 10 minutes from March 2007 to September 2011 in each of the four streams. Mean turbidity was measured using an instream nephelometer, which measures the scatter of a focused light beam by suspended solids. We evaluated multiple years of simulated data under each scenario (i.e., baseline, forest harvest, climate change, combined) for each stream. Because we had observations from only 2007–2011, we randomly selected the environmental regimes from one of those initial years every year for 67 years (a duration chosen for computational reasons). This ordering of years became the baseline scenario and was modified to create all alternative scenarios. Once the ordering of years was set for a scenario, then daily values of each environmental regime were read into the model. After we ran our scenarios, we ignored the first four years of data from all scenarios to eliminate initialization effects [20, 21]. Accordingly, we applied the changes in stream temperature and flow from scenarios beginning in year five, which became year one after initialization effects were eliminated. Therefore, we used a total of 63 years of results to analyze each scenario.

### Responses of trout

We analyzed output produced every 30 simulated days from five replicate model runs for each scenario. We averaged population biomass (g), growth (cm/month; fork length), and survival (proportion of total that survived in each age class) by season each year based on the five replicate model runs (S1 File). Seasons include summer (July, August) and winter (January, February). Age classes during winter were restricted to ages 1 and older because the model assigns an additional year to each trout on January 1^st^ every year (in keeping with convention in fisheries biology), thus there are no age-0 trout in winter. For biomass, growth, and survival, we used average responses of each trout age class (ages 0, 1, 2, 3+). Timing of fry emergence was evaluated as the median emergence date (Julian day at which half the age 0 fish had emerged), estimated using MATLAB and Statistics Toolbox Release 2012 (The MathWorks, Inc. Natick, Massachusetts, US; see code in [40]).

### Statistical analyses

We evaluated population biomass (g), growth (cm/month; fork length), and survival (proportion of total that survived in each age class) by season each year over the five replicate model runs. To provide an overall view of differences among streams and scenarios, we evaluated summer and winter responses of median biomass, growth, and survival for each age class for each scenario (baseline, forest harvest, climate change, and combined) across all 63 simulation years using non-parametric Kruskal-Wallis one-way analysis of variance on ranks. In cases of statistically significant differences (alpha ≤ 0.05) indicated by these analyses, we evaluated pairwise comparisons among the four scenarios using a non-parametric Tukey’s test on ranks. We focused these tests on deviations of responses from a stream’s specific baseline scenario to responses observed for that stream related to forest harvest, climate change, and combined scenarios (i.e., baseline vs. other scenarios). When the scenario for a stream deviated significantly from its own baseline, we also examined the direction and magnitude of the observed differences.

Annual trends of both the median date of fry emergence and difference in magnitude of biomass of scenario compared to baseline for each stream were evaluated using the Mann-Kendall test [41–42]. This rank-based test is robust to outliers and non-linear trends [43–44]. The Mann-Kendall trend values were corrected for serial correlation [45–46] using the package FUME for R [47]. We evaluated annual trends of date of fry emergence and difference in magnitude of the scenario of interest and baseline over three timescales, including up to year 20, from year 20 to year 63, and over the 63-year duration of the study for all scenarios. By examining the trend up to year 20, we capture the time period when forest harvest may have the greatest impact and in a timeframe that a trend signal could be detected.

The question of whether total biomass of trout in summer under each scenario differed from baseline values at each stream was examined using a Wilcoxon signed rank paired test with continuity correction using the package STATS for R. In addition, within the simulated scenarios of forest harvest and climate change, we tested the individual influences of stream temperature and flow on differences in biomass of trout in summer relative to baseline values. We tested the null hypothesis that the median of the difference between baseline and the other scenarios was symmetric about 0. A nonparametric confidence interval and an estimator for the pseudo-median for the difference between the scenarios was also computed [48–49]. The Kruskal-Wallis, Mann-Kendall analyses, and Wilcoxon signed rank analyses were analyzed using software R ver. 2.15.1 [50].

## Results and Discussion

### Idiosyncratic responses in trout emerge from local variability among streams

**Fig 2:**
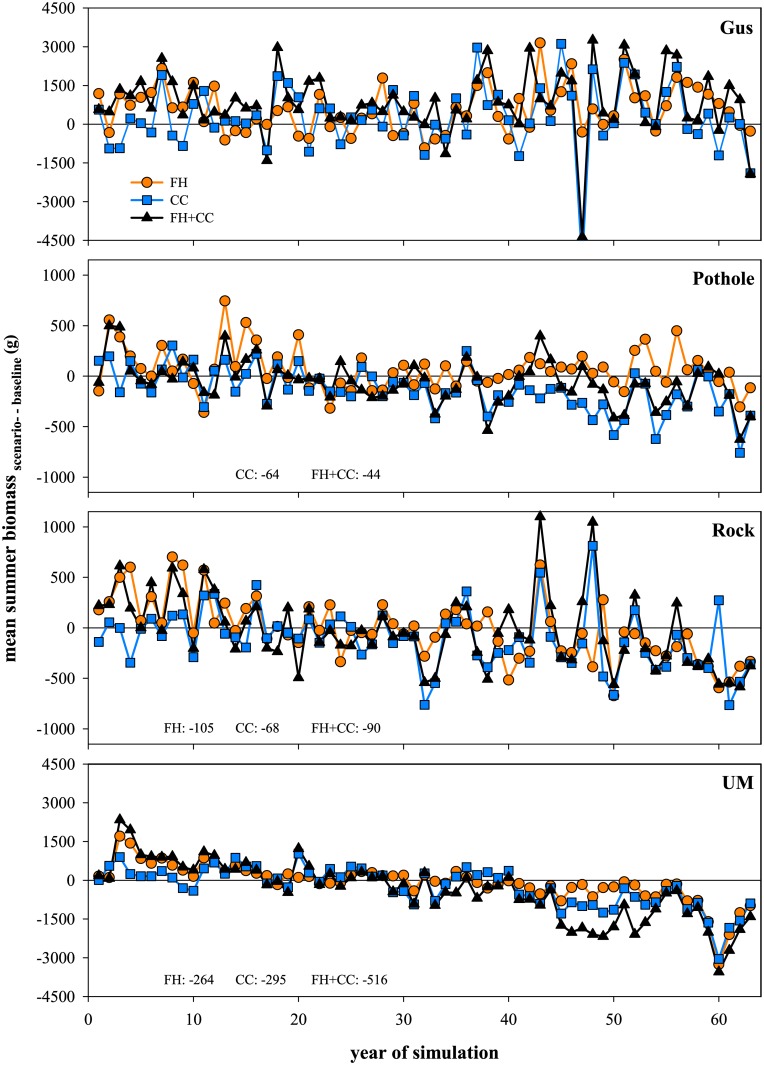
Differences in Total Summer Biomass of Trout between Scenarios and Baseline. Difference in mean total summer biomass (g) from the five replicated simulations over time for each scenario of forest harvest (FH), climate change (CC), and their combined effects (FH+CC) compared to baseline, in Gus Creek, Pothole Creek, Rock Creek, and Upper Mainstem (UM). Scenarios include manipulations of stream temperature and flow regimes (see methods narrative for detail). Only significant trends (P < 0.05) for the entire study period have been numerically shown with the slope of the trend (g/decade).

**Fig 3:**
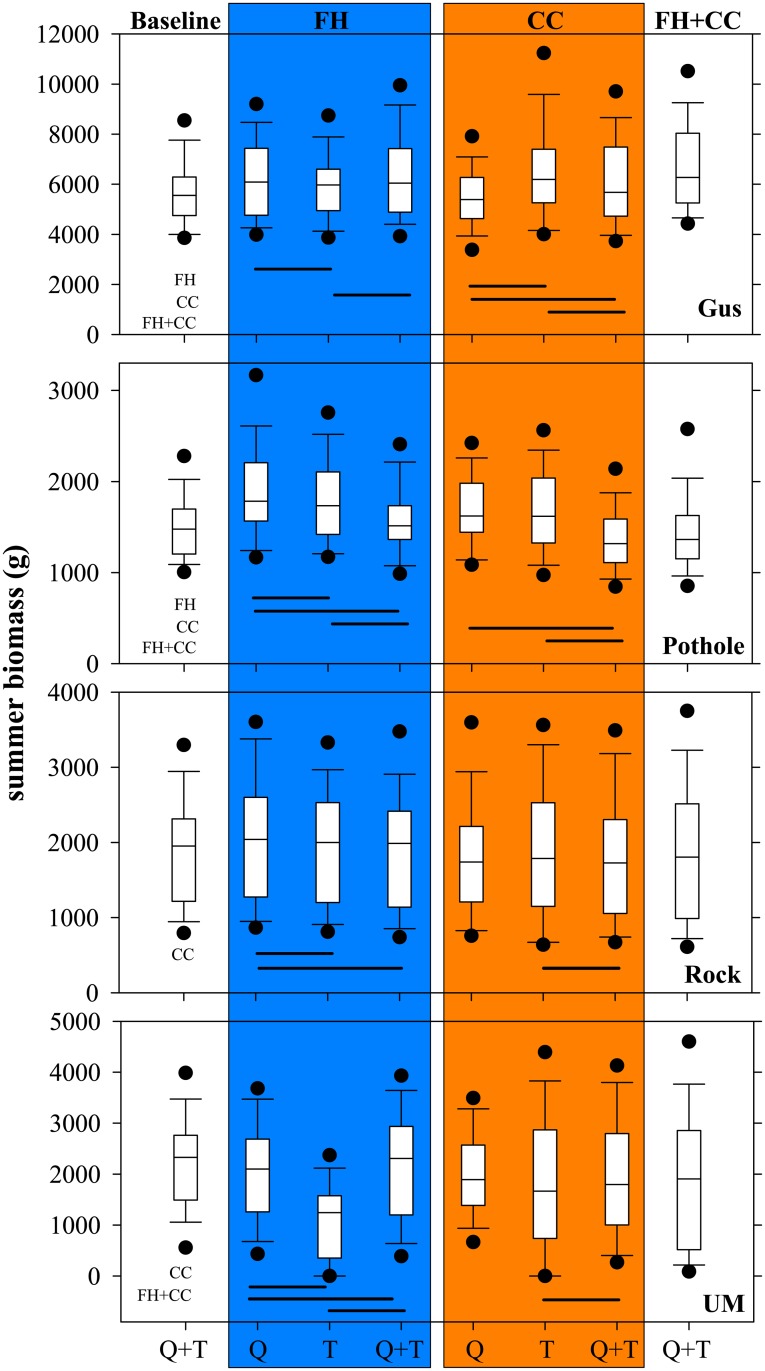
Influence of Flow and Temperature on Trout Biomass within each Scenario. Boxplots of mean total summer biomass (g) of trout in Gus Creek, Pothole Creek, Rock Creek, and Upper Mainstem (UM) from five replicate simulations over the entire study period. Each boxplot incorporates 63 data points of the mean of every year’s summer biomass per scenario. Gray boxes represent pairwise comparisons of the influence of flow (Q), stream temperature (T), and both (Q+T) within each scenario of forest harvest (FH) and climate change (CC). Baseline and the combined scenarios (FH+CC) are shown for reference. Scenarios include manipulations of stream temperature and flow regimes (see methods narrative for detail). Significant pairwise differences are shown by a horizontal black line (P < 0.05). Significant differences between baseline and each scenario are noted. The point above or below each boxplot corresponds to the 5^th^ and 95^th^ percentile.

Our simulations show that local variability in stream conditions influences the idiosyncratic responses of trout to projections of forest harvest and climate change across streams (Table 1). For example, differences in total biomass of trout in summer between baseline and forest harvest and climate change scenarios indicated diminishing trends or no change over time depending on stream conditions (Fig 2 and S2 Table). In addition, when the effects of either temperature or flow were evaluated separately and compared to their combined effects, idiosyncratic responses of trout biomass were generally found across streams for each scenario (Fig 3 and S3 Table). For example, under the climate change scenario, the separate effect of flow resulted in lower biomass than the separate effect of temperature in Gus Creek, but the opposite was seen in Pothole Creek, Rock Creek, and Upper Mainstem. Similar mixed responses were also seen under the forest harvest scenario, where the combined effect of temperature and flow after forest harvest resulted in lower biomass in Upper Mainstem when compared to separate effect of flow, whereas in Rock and Pothole Creeks their combined effect resulted in higher biomass and no difference in Gus Creek.

**Table 1:** Summer Survival, Growth, and Biomass by Age Class.

		age 0			age 1			age 2			age 3+		
variable	stream	FH	CC	FH+CC	FH	CC	FH+CC	FH	CC	FH+CC	FH	CC	FH+CC
survival	Gus	-	-50%	-55%	-	-	-	-	-	-	-	-	-
	Pothole	-	-	-	-	-13%	-9%	-	-	-	-	-	-
	Rock	-	-18%	-16%	-	-25%	-23%	-	-7%	-19%	-	-	-
	UM	-	-20%	-14%	-	-	-	-	-	-	-	-	-
growth	Gus	-	28%	28%	-	-	-	-	-	-	-	-	-
	Pothole	-	11%	11%	-	60%	18%	-	-	-	-	-52%	-
	Rock	3%	-	-	58%	198%	252%	-	-	-	-	-	-
	UM	9%	-	-	-	-49%	-60%	-	-	-	-	-	-
biomass	Gus	13%	35%	-20%	-	-22%	-44%	-	-	-	15%	-20%	-8%
	Pothole	14%	10%	17%	-	-	-5%	-	-21%	-10%	-	-13%	-
	Rock	-	25%	23%	-5%	-	-	-	-54%	-34%	-	-79%	-61%
	UM	-	121%	16%	-	-25%	-32%	-	-38%	-38%	-	-26%	-36%

Difference in median summer survival (proportion of total in that age class that survive), growth (cm/month), and biomass (g) of median values for four age classes (ages 0, 1, 2, 3+) of trout in relation to baseline for forest harvest (FH), climate change (CC), and combined (FH + CC) scenarios in four modeled streams over 63 years. Streams include Gus Creek, Pothole Creek, Rock Creek, and Upper Mainstem (UM). Scenarios include manipulations of stream temperature and flow regimes (see methods for detail). The magnitude of change for each scenario relative to baseline is an average of five replicate simulations and is calculated as: [(median scenario − median baseline)/median baseline]*100. Summer is July and August. Responses were analyzed using Kruskal-Wallis one-way analysis of variance on ranks. Negative values indicate that the values for the response of the scenario of interest is lower than baseline values for that stream and positive values indicate that it is higher than baseline. Only significant responses are shown (alpha ≤ 0.05).

Although our modeled streams are physically similar in many respects (e.g., similar mean summer flows for three of them, similar watershed area for three of them, similar summer velocities for three of them, S1 Table), there are some notable differences among them that mediate the sensitivity of trout to forest harvest and climate change. Among our four study streams, trout in Gus Creek often showed unique responses indicating an overall resistance to change due to differences in physical habitat conditions compared to the other streams. Gus Creek, for its watershed area, supports a relatively larger wetted area and deeper pools in summer, a time period when stresses on fish are high [51] and both growth [52] and survival are minimal [53]. Our findings are in agreement with a sensitivity analyses performed on these study streams that highlights the importance of habitat availability in driving growth and survival of trout during seasonal low-flow [33]. More broadly, studies across the Pacific Northwest report localized responses of stream temperature and flow to either climate change or forest harvest, due to contrasting features in streams (e.g, for temperature [26, 54]; for flow [27, 38]). Local responses of fish to forest harvest have also been commonly described (see reviews [55–56]). Although our results contrast with other research of fish responses to climate change, which typically reports relatively uniform responses by stream-fish across landscapes [57–60], and for a wide-range of other biota as well [14, 61–63], more recently, increasing attention has been drawn to the role of local variability in physical habitat as an important player in mediating the effects of climate change [8, 16, 18, 64].

### Separate projections differ from scenarios incorporating combined effects

Simulated responses of trout from projections of forest harvest and climate change scenarios differed substantially from scenarios incorporating their combined effects (Table 2 and S2 Fig). Although we observed cases where the combined effects of forest harvest and climate change exceeded the additive influences of each, we commonly noted interactions where the effects from the combined scenario were less than the additive effects from separate scenarios. For example, over the entire study period in Pothole Creek, forest harvest increased biomass and the combined scenario decreased biomass, but less so than the climate change scenario alone. In general in ecological settings, although it may be expected that multiple stressors should interact synergistically, antagonistic responses are more frequently observed [65–66]. In some cases we simulated here, forest harvest countered the effects of climate change. Specifically, the relative biomass of trout in Gus Creek increased under the combined scenario compared to climate change alone because increases in low-flow discharge associated with forest harvest offsets climate-driven declines in flows. Such findings are, however, highly context dependent and they depend on how future climate effects are realized. For example, trout responses to climate change could be more dramatic than we have modeled here if we had modeled longer simulations or when natural environmental conditions approach or exceed stressful thresholds for trout, especially during seasonal low flow. For example, if stream temperature continues to warm towards levels that are stressful to trout, increasing summer flows from forest harvest may no longer effectively offset warming temperatures.

**Table 2:** Pairwise Comparisons for Differences in Total Summer Biomass between Scenario and Baseline.

		1^st^ harvest				2^nd^ harvest				entire 63 years			
stream	scenario	Δ (g)	95% CI	V	p-value	Δ (g)	95% CI	V	p-value	Δ (g)	95% CI	V	p-value
Gus	FH	576	172, 928	178	0.26	1000	560, 1438	200	**<0.001**	542	315, 482	1646	**<0.001**
	CC	218	-203, 713	136	**0.005**	519	-172, 1192	148	0.12	285	23, 578	1328	**0.03**
	FH+CC	862	547, 1328	195	**<0.001**	1162	247, 1774	181	**<0.001**	852	587, 1125	1803	**<0.001**
Pothole	FH	173	37, 300	174	**0.008**	80	32, 153	176	**0.006**	51	8, 103	1343	**0.02**
	CC	23	-61, 122	123	0.52	-232	-331, -138	5	**<0.001**	-125	-176, -70	400	**<0.001**
	FH+CC	43	-41, 166	131	0.35	-77	-180, 11	61	0.11	-63	-113, 14	650	**0.01**
Rock	FH	211	63, 334	179	**0.004**	-206	-306, -61	35	**0.007**	-25	-95, 54	892	0.43
	CC	-10	-94, 99	96	0.76	-219	-339, -10	52	**0.05**	-119	-190, -48	543	**0.001**
	FH+CC	140	-3, 293	153	0.08	-134	-300, -93	72	0.23	-50	-143, 40	863	0.32
UM	FH	474	267, 715	205	**<0.001**	-452	-704, -243	0	**<0.001**	-15	560, 1438	970	0.80
	CC	298	87, 504	175	**0.007**	-881	-1082, -660	0	**<0.001**	-213	-395, -16	698	**0.03**
	FH+CC	658	376, 955	197	**<0.001**	-1410	-1865, 1071	0	**<0.001**	-341	-658, -41	660	**0.02**

Pairwise comparisons of total biomass (g) of trout in summer for forest harvest (FH), climate change (CC), and combined (FH + CC) scenarios compared to baseline in modeled streams, including Gus Creek, Pothole Creek, Rock Creek, and Upper Mainstem (UM). Values of summer biomass by year were averaged for five replicate simulations and were analyzed using Wilcoxon signed rank test (V) with continuity correction resulting in a pseudomedian of difference between scenario and baseline (Δ) for the 1^st^ harvest period, 2^nd^ harvest period, and the entire study period. Scenarios include manipulations of stream temperature and flow regimes (see Methods for details). Significant p-values in bold (alpha ≤ 0.05) represent increasing or decreasing magnitudes in comparison to baseline.

### Climate change influences trout more than forest harvest

**Fig 4:**
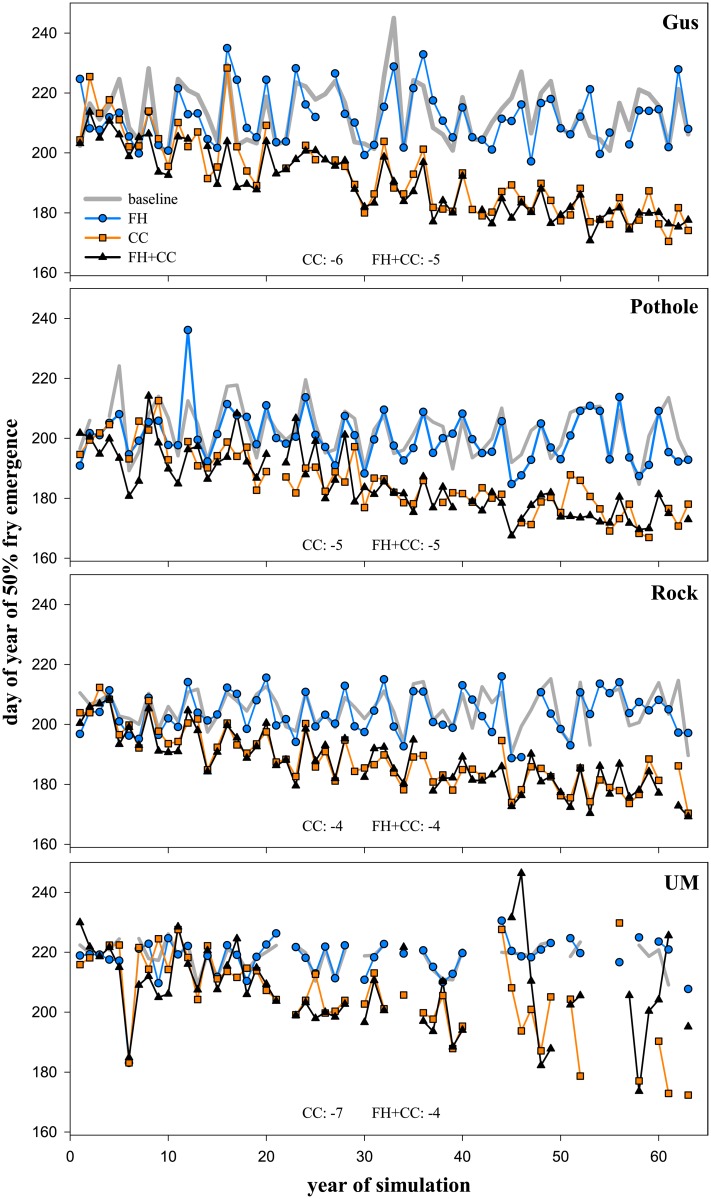
Trends in Fry Emergence of Trout across Scenarios. DOY from five replicate simulations when median number of modeled fry had emerged over time in Gus Creek, Pothole Creek, Rock Creek, and Upper Mainstem (UM). Scenarios include manipulations of stream temperature and flow regimes (see methods narrative for detail). Only significant trends (P < 0.05) over time are listed and include the slope of the trend (days per decade). Negative values represent early fry emergence. Gaps in data are due to years with no fry emergence because model thresholds for spawning, egg development, or emergence were not met.

Our modeled findings indicate that climate change alone influences trout more strongly and consistently across our study streams than forest harvest. Climate change led to earlier fry emergence (Fig 4 and S4 Table), reductions in biomass of older trout, and increased biomass of young-of-year (Table 1 and S5 Table), but these changes did not consistently translate into trends toward reduced biomass over time (Fig 2 and S2 Table). They did, however, collectively lead to lower biomass relative to baseline conditions (Table 2 and S2 Fig). Reductions in biomass of older trout come from both smaller-sized and reduced number of older individuals. Although other studies have similarly predicted a decrease in body size of individuals with increasing temperature from climate change, underlying mechanisms have been difficult to identify [67–68]. Because it is not clear how climate change will influence the amount of drift or benthic food for trout, in our simulations, we assume that food availability does not change with temperature [20–21]. Increased temperature due to climate change leads to higher metabolism in trout, but because food intake does not change trout do not grow. Under such circumstances, they either starve or they choose riskier habitat with higher predation risk, both of which lead to lower abundances. We also noted increased biomass in young-of-year in most of the cases (see empirical evidence in [67]) due to reduced intraspecific competition.

Forest harvest, in contrast, had much less effect on growth and survival than climate change (Table 1 and S5 Table), and mixed responses for biomass and trends in biomass over time (increases, decreases, or no change; Fig 2, S2 Fig, Table 2 and S2 Table). It is not surprising that trout responses were minimal across streams in response to projections of forest harvest because this stressor acts as a pulsed event (where temperature and flow returned to baseline conditions within 1–2 decades). It is worth noting, however, that we only considered effects of forest harvest on stream temperature and flow, and that other influences could also have important implications. For example, changes in stream habitat associated with loss (or addition) of large wood as a result of forestry practices and stream restoration [69] and changes to trout food supply following harvest [51]. Therefore, consideration of these additional factors might be expected to further reinforce the importance of the specific array of local conditions mediating trout responses to multiple stressors. Additionally, we found the types of effects realized from forest harvest were important in two streams (Rock Creek and Upper Mainstem; Table 2) with the net effect switching from increasing biomass after the first harvest to decreasing biomass after the second harvest. The net effect switches in these streams, not because of forest harvest *per se*, but rather because trout in these streams were less able to cope with instream changes from either stressor (second perturbation of forest harvest or climate change) after about 30 years.

### Most visible response of trout to change was a phenology metric

The most visible and consistent response from our simulations was earlier fry emergence driven by climate change (Fig 4 and S4 Table), whereas demographic responses of survival, growth, or biomass were not detectable and inconsistent across scenarios and streams (Fig 2 and S2 Fig). Under the climate change and combined scenarios, there were consistent trends toward earlier fry emergence for all streams over time. Over the entire study period, median timing of fry emergence was earlier by 4 to 7 days/decade for climate change, but it was earlier by only 4 to 5 days days/decade for the combined scenario due to the trend towards later emergence generally observed with forest harvest. Early fry emergence under the climate change scenario largely results from warmer stream temperature that accelerates the development of eggs across streams over time. Similarly, a lab study shows that fry emergence can be delayed due to increased temporal variance in temperature regimes [70]. Collectively, we highlight the importance of magnitude and variability of temperature regimes on emergence. Studies of salmonid fishes in other systems have shown that earlier emergence timing can negatively influence survival of subsequent life stages [71]. In general, changes in phenology are a reliable indicator of climate change on diverse taxa [61, 72]. Although we find a coherent change in fry emergence across streams for the climate change scenario, this does not consistently translate into changes in trends of trout biomass or other demographic responses.

Demographic responses of survival, growth, or biomass depended on scenarios and streams (Fig 2 and S2 Fig). Most of the changes in trout demography occurred for climate change and combined scenarios, rather than for forest harvest in both summer and winter (Table 1 and S5 Table). Overall, survival and growth of age-0 and age-1 trout frequently responded to changes linked to forest harvest and climate change more than older age classes. Age-0 trout exhibited increased survival over summer for all scenarios because earlier fry emergence (Fig 4 and S3 Table) increased recruitment, with the exception of Upper Mainstem which is similar to an observational study [71]. Age-0 trout exhibited increased growth over summer in the climate change and combined scenarios compared to baseline (except for Upper Mainstem and Rock Creek). Age-0 trout showed increased biomass for climate change and combined scenarios compared to baseline (except Gus Creek under the combined scenario). During both seasons, age-1 trout showed mixed responses in growth and biomass for climate change and combined scenarios compared to baseline across streams. In contrast, biomass responded more uniformly across all age classes. Age-2 and age-3+ trout most consistently showed reductions in biomass for climate change and combined scenarios compared to baseline across streams. These idiosyncratic responses for demography may have been driven by the complex interplay of underlying factors, including density-dependence, specific requirements of environmental conditions at each age class (habitat), and variability in demography and the environment [73]. In our simulations, we held most variables constant and equal across streams, except for local differences in physical habitat conditions and variability in environmental regimes among streams highlighting habitat (depth and area of available habitat) as the key driver [33]. The lack of coherence among responses of demography of trout has practical consequences because biological responses commonly quantified in field studies (survival, growth, and biomass) may not be able to consistently indicate the effects of land use and climate change [74].

## Conclusions

In summary, results of this work reveal a diverse array of responses to identical changes in stream temperature and flow projected from forest harvest and climate change on four neighboring streams within the same headwater catchment. We infer that it is important to identify the array of key features at the local scale that will allow fish to resist environmental change. If we had simulated higher changes in magnitude over longer time periods, we might have expected to see greater coherence in responses. For example, recent work has shown that as the effects of climate change intensify, uncertainty regarding species’ responses to climate become less important, relative to our uncertainty about climate itself [75]. As the growing importance of both land use and climate change becomes more broadly accepted and acted upon within the policy arena, there is an increasing need for approaches to clearly attribute biological responses to such changes. Although there are conditions where it is relatively straightforward to diagnose the influences of land use, climate change, or other stressors on biological responses, there are many other contexts where attribution will be substantially more complex and less certain. As this and other recent studies have shown, models based heavily in field data that are capable of addressing such complexity can allow a multitude of useful insights into interactions among local processes. Our findings provide support for continuing this line of inquiry and reveal the importance of disentangling the role of local variability in physical habitat conditions in streams under different land uses and a changing climate.

## Supporting Information

S1 Fig Map of Trask Watershed, OR, USA, with Photos of Field Sites. Sub-basin of Pothole Creek is purple, Gus Creek is light blue, Upper Mainstem Trask is yellow, and Rock Creek is brown. Modeled stream reaches representing actual field sites are orange.
(PNG)

S2 Fig Summer Biomass of Trout over Time. Mean total summer biomass (g) from five replicate simulations for all trout ages grouped together over time for each scenario of forest harvest (FH), climate change (CC), and their combined effects (FH+CC) compared to baseline, in Gus Creek, Pothole Creek, Rock Creek, and Upper Mainstem (UM). Scenarios include manipulations of stream temperature and regimes (see methods narrative for detail). Each data point represents one year of the simulation run. Line represents 1:1 ratio between the scenario and baseline. Only significant comparisons (P < 0.05) are shown with values that correspond to pseudomedian of differences in biomass (g) between scenario and baseline.
(JPG)

S1 File This file contains input files for inSTREAM and output files used to generate Figures and tables. Calibration calculations and values for environmental regimes can be found in [33].
(XLSX)

S1 Table Characteristics of Study Streams. Description of environmental (ENVR) regimes, instream habitat features, and calibration values for Gus Creek, Pothole Creek, Rock Creek, and Upper Mainstem Trask (UM). ENVR regimes display average values with standard deviation values in parentheses for data collected from March 2007 to September 2011. Winter is January and February and summer is July and August. Distance to hiding cover, availability of velocity shelter, spawning gravel, velocity by season, and depth by season are averaged values (total/no. of cells in stream). Higher distance to hiding cover values represent less overall hiding cover availability. Velocity shelter and spawning gravel are each estimated as a percentage of cell area with that characteristic.
(DOCX)

S2 Table Trends of Differences in Summer Biomass of Trout between Scenarios and Baseline. Trends of differences in magnitude of total biomass (g) of trout in summer for forest harvest (FH), climate change (CC), and combined (FH + CC) scenarios compared to baseline in modeled streams, including Gus Creek, Pothole Creek, Rock Creek, and Upper Mainstem (UM). Annual trends in total biomass (g/decade) were averaged across five replicate simulations. They were analyzed using the Mann-Kendall test and p-values were corrected for serial correlation for 1^st^ harvest, 2^nd^ harvest, and the entire study period. Scenarios include manipulations of stream temperature and flow regimes (see Methods for details). Significant p-values in bold (alpha ≤ 0.05) represent increasing or decreasing trends of magnitude in comparison to baseline. Magnitude is Sen slope (g/decade) over time.
(DOCX)

S3 Table Pairwise Comparisons of Summer Biomass of Trout. Pairwise comparisons of total summer biomass (g) of trout for baseline, forest harvest (FH), climate change (CC), and combined (FH + CC) scenarios in Gus Creek, Pothole Creek, Rock Creek, and Upper Mainstem (UM) for the entire study period. Individual influences from stream temperature and flow were considered for single scenarios of FH and CC. Scenarios include manipulations of stream temperature and flow regimes (see Methods for details). Values of summer biomass by year were averaged for five replicate simulations and were analyzed using Wilcoxon signed rank test (V) with continuity correction resulting in a pseudomedian of difference between group 1 and group 2 (Δ). Significant p-values in bold (alpha ≤ 0.05).
(DOCX)

S4 Table Trends in Fry Emergence of Trout across Scenarios. Median DOY of fry emergence of trout for forest harvest (FH), climate change (CC), and combined (FH + CC) scenarios in four modeled streams, including Gus Creek, Pothole Creek, Rock Creek, and Upper Mainstem (UM). Annual trends in fry emergence (days/decade) were averages of the five replicate simulations and were analyzed using the Mann-Kendall test and p-values were corrected for serial correlation for dates of the 1^st^ harvest, 2^nd^ harvest, and the entire study period. Scenarios include manipulations of stream temperature and flow regimes (see Methods for details). Significant p-values in bold (alpha ≤ 0.05) represent increasing or decreasing trends. Magnitude is the Sen slope (days/decade) over time.
(DOCX)

S5 Table Pairwise Comparisons of Winter Survival, Growth, and Biomass by Trout Age Class. Pairwise comparisons of winter survival (proportion of total in that age class that survive), growth (cm/month), and biomass (g) for three age classes (ages 1, 2, 3+) of trout in relation to baseline for forest harvest (FH), climate change (CC), and combined (FH + CC) scenarios in four modeled streams over 63 years. Streams include Gus Creek, Pothole Creek, Rock Creek, and Upper Mainstem (UM). Scenarios include manipulations of stream temperature and flow regimes (see methods for detail). The magnitude of change for each scenario relative to baseline is an average of five replicate simulations and is calculated as: [(median scenario − median baseline)/median baseline]*100. Winter is January and February. Age classes during winter were restricted to ages 1+ because the model assigns an additional year to each trout on January 1^st^ every year, thus there are no age 0 trout in winter. Responses were analyzed using Kruskal-Wallis one-way analysis of variance on ranks. Negative values indicate that the values for the response of the scenario of interest is lower than baseline values for that stream and positive values indicate that it is higher than baseline. Only significant responses are shown (alpha ≤ 0.05).
(DOCX)
