# Supplementary figures and images for: Local Variability Mediates Vulnerability of Trout Populations to Land Use and Climate Change
Source: PLoS One. 2015 Aug 21;10(8):e0135334. doi: 10.1371/journal.pone.0135334 (PMC4546676; doi:10.1371/journal.pone.0135334)

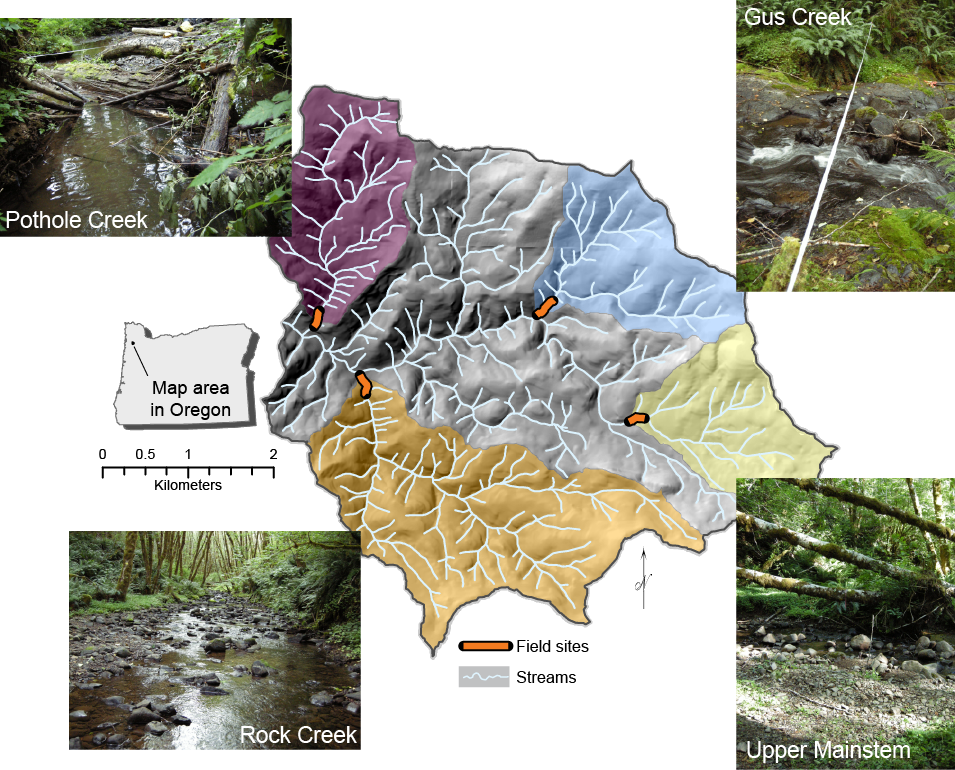

Supplement: S1 Fig — Sub-basin of Pothole Creek is purple, Gus Creek is light blue, Upper Mainstem Trask is yellow, and Rock Creek is brown. Modeled stream reaches representing actual field sites are orange. (PNG) [file pone.0135334.s001.png]

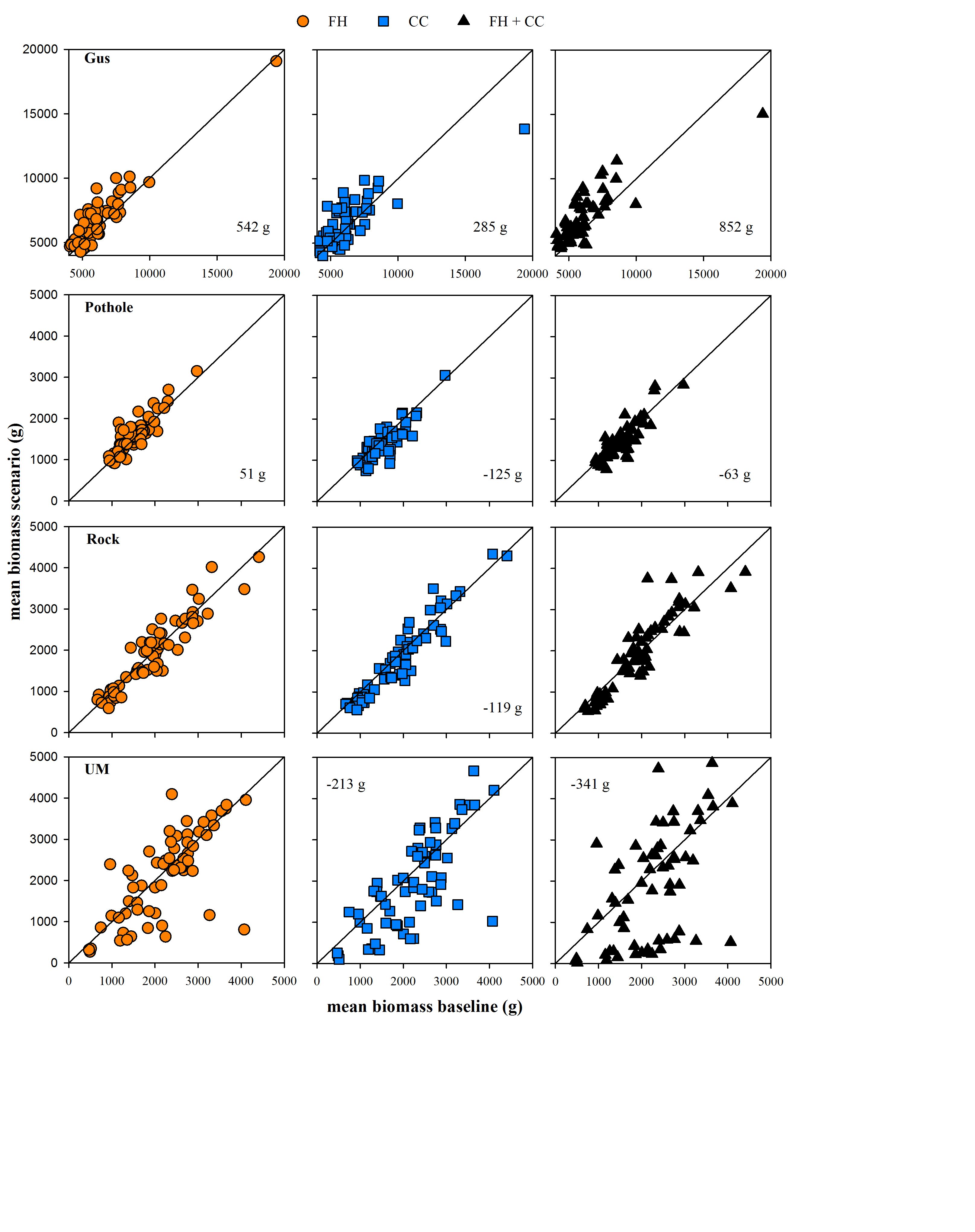

Supplement: S2 Fig — Mean total summer biomass (g) from five replicate simulations for all trout ages grouped together over time for each scenario of forest harvest (FH), climate change (CC), and their combined effects (FH+CC) compared to baseline, in Gus Creek, Pothole Creek, Rock Creek, and Upper Mainstem (UM). Scenarios include manipulations of stream temperature and regimes (see methods narrative for detail). Each data point represents one year of the simulation run. Line represents 1:1 ratio between the scenario and baseline. Only significant comparisons (P < 0.05) are shown with values that correspond to pseudomedian of differences in biomass (g) between scenario and baseline. (JPG) [file pone.0135334.s002.jpg]
