# Supplementary material for: Local Variability Mediates Vulnerability of Trout Populations to Land Use and Climate Change
Source: PLoS One. 2015 Aug 21;10(8):e0135334. doi: 10.1371/journal.pone.0135334 (PMC4546676; doi:10.1371/journal.pone.0135334)
Supplement: S1 Table — Description of environmental (ENVR) regimes, instream habitat features, and calibration values for Gus Creek, Pothole Creek, Rock Creek, and Upper Mainstem Trask (UM). ENVR regimes display average values with standard deviation values in parentheses for data collected from March 2007 to September 2011. Winter is January and February and summer is July and August. Distance to hiding cover, availability of velocity shelter, spawning gravel, velocity by season, and depth by season are averaged values (total/no. of cells in stream). Higher distance to hiding cover values represent less overall hiding cover availability. Velocity shelter and spawning gravel are each estimated as a percentage of cell area with that characteristic. (DOCX) [file pone.0135334.s004.docx]

**S1 Table. Characteristics of Study Streams.**

| Model input | Factor |  |  |  | |
| --- | --- | --- | --- | --- | --- |
|  |  | Gus | Pothole | Rock | UM |
| ENVR | Winter stream temp (^°^C) | 4.8(1.4) | 6.4(0.9) | 5.8(1.2) | 4.7(1.1) |
| regimes | Winter stream flow (m^3^/s) | 0.51(1.22) | 0.37(0.48) | 0.73(0.74) | 0.26(0.24) |
|  | Winter turbidity (NTU) | 11.6(39.6) | 17.6(52.1) | 7.7(18.6) | 11.8(17.3) |
|  | Summer stream temp (^°^C) | 11.1(1.3) | 10.9(0.7) | 11.3(1.0) | 10.5(1.1) |
|  | Summer stream flow (m^3^/s) | 0.03(0.02) | 0.03(0.00) | 0.03(4.10) | 0.04(0.02) |
|  | Summer turbidity (NTU) | 9.9(8.1) | 3.6(2.1) | 4.9(0.0) | 10.5(8.9) |
|  |  |  |  |  |  |
| Instream | Watershed area (ha) | 302.1 | 325.4 | 667.6 | 293.2 |
| habitat | Wetted area in summer (ha) | 0.15 | 0.101 | 0.142 | 0.079 |
| features | Elevation (m) | 469 | 324 | 337 | 609 |
|  | Distance to hiding cover (m) | 2.40 | 2.30 | 1.75 | 1.06 |
|  | Velocity shelter | 0.40 | 0.36 | 0.88 | 0.32 |
|  | Spawning gravel | 0.14 | 0.06 | 0.10 | 0.17 |
|  | Winter velocity (m/s) | 0.42 | 0.32 | 0.40 | 0.20 |
|  | Winter depth (m) | 0.54 | 0.29 | 0.63 | 0.33 |
|  | Summer velocity (m/s) | 0.18 | 0.16 | 0.19 | 0.09 |
|  | Summer depth (m) | 0.23 | 0.09 | 0.10 | 0.15 |
|  | Cells (no. per stream) | 35 | 35 | 31 | 32 |
|  |  |  |  |  |  |
| Calibration | Drift food (g) | 1.5x10-9 | 1.5x10-9 | 1.5x10-9 | 6x10-10 |
|  | Benthic food (g) | 5x10-7 | 5x10-7 | 5x10-7 | 8x10-7 |
|  | Aquatic predation | 0.900 | 0.900 | 0.900 | 0.960 |
|  | Terrestrial predation | 0.990 | 0.990 | 0.990 | 0.985 |

Description of environmental (ENVR) regimes, instream habitat features, and calibration values for Gus Creek, Pothole Creek, Rock Creek, and Upper Mainstem Trask (UM). ENVR regimes display average values with standard deviation values in parentheses for data collected from March 2007 to September 2011. Winter is January and February and summer is July and August. Distance to hiding cover, availability of velocity shelter, spawning gravel, velocity by season, and depth by season are averaged values (total/no. of cells in stream). Higher distance to hiding cover values represent less overall hiding cover availability. Velocity shelter and spawning gravel are each estimated as a percentage of cell area with that characteristic.
