# Supplementary material for: Local Variability Mediates Vulnerability of Trout Populations to Land Use and Climate Change
Source: PLoS One. 2015 Aug 21;10(8):e0135334. doi: 10.1371/journal.pone.0135334 (PMC4546676; doi:10.1371/journal.pone.0135334)
Supplement: S2 Table — Trends of differences in magnitude of total biomass (g) of trout in summer for forest harvest (FH), climate change (CC), and combined (FH + CC) scenarios compared to baseline in modeled streams, including Gus Creek, Pothole Creek, Rock Creek, and Upper Mainstem (UM). Annual trends in total biomass (g/decade) were averaged across five replicate simulations. They were analyzed using the Mann-Kendall test and p-values were corrected for serial correlation for 1st harvest, 2nd harvest, and the entire study period. Scenarios include manipulations of stream temperature and flow regimes (see Methods for details). Significant p-values in bold (alpha ≤ 0.05) represent increasing or decreasing trends of magnitude in comparison to baseline. Magnitude is Sen slope (g/decade) over time. (DOCX) [file pone.0135334.s005.docx]

**S2 Table. Trends of Differences in Summer Biomass of Trout between Scenarios and Baseline.**

|  |  | 1^st^ harvest | | | 2^nd^ harvest | | | entire 63 years | | |
| --- | --- | --- | --- | --- | --- | --- | --- | --- | --- | --- |
| stream | scenario | tau | p-value | slope | tau | p-value | slope | tau | p-value | slope |
| Gus | FH | -0.29 | 0.07 | -651 | 0.11 | 0.41 | 202 | 0.06 | 0.50 | 59 |
|  | CC | 0.23 | 0.16 | 763 | -0.06 | 0.72 | -147 | 0.04 | 0.63 | 59 |
|  | FH+CC | -0.09 | 0.58 | -172 | -0.14 | 0.42 | -413 | -0.04 | 0.60 | -21 |
| Pothole | FH | 0.01 | 0.97 | -59 | -0.11 | 0.54 | -23 | -0.08 | 0.35 | -12 |
|  | CC | -0.06 | 0.43 | -26 | -0.07 | 0.67 | -29 | -0.41 | **<0.001** | -64 |
|  | FH+CC | -0.11 | 0.54 | -90 | -0.11 | 0.54 | -97 | -0.26 | **<0.001** | -44 |
| Rock | FH | -0.40 | **0.01** | -242 | -0.26 | 0.11 | -173 | -0.49 | **<0.001** | -105 |
|  | CC | 0.03 | 0.87 | 20 | -0.14 | 0.14 | -94 | -0.30 | **<0.001** | -68 |
|  | FH+CC | -0.38 | **0.02** | -262 | -0.38 | **<0.001** | -248 | -0.36 | **<0.001** | -90 |
| UM | FH | -0.41 | **0.02** | -520 | -0.27 | 0.23 | -247 | -0.67 | **<0.001** | -264 |
|  | CC | 0.01 | 0.97 | -69 | -0.18 | 0.28 | -148 | -0.52 | **<0.001** | -295 |
|  | FH+CC | -0.31 | 0.06 | -806 | -0.14 | 0.42 | -128 | -0.68 | **<0.001** | -516 |

Trends of differences in magnitude of total biomass (g) of trout in summer for forest harvest (FH), climate change (CC), and combined (FH + CC) scenarios compared to baseline in modeled streams, including Gus Creek, Pothole Creek, Rock Creek, and Upper Mainstem (UM). Annual trends in total biomass (g/decade) were averaged across five replicate simulations. They were analyzed using the Mann-Kendall test and p-values were corrected for serial correlation for 1^st^ harvest, 2^nd^ harvest, and the entire study period. Scenarios include manipulations of stream temperature and flow regimes (see Methods for details). Significant p-values in bold (alpha ≤ 0.05) represent increasing or decreasing trends of magnitude in comparison to baseline. Magnitude is Sen slope (g/decade) over time.
