# Supplementary material for: Local Variability Mediates Vulnerability of Trout Populations to Land Use and Climate Change
Source: PLoS One. 2015 Aug 21;10(8):e0135334. doi: 10.1371/journal.pone.0135334 (PMC4546676; doi:10.1371/journal.pone.0135334)
Supplement: S3 Table — Pairwise comparisons of total summer biomass (g) of trout for baseline, forest harvest (FH), climate change (CC), and combined (FH + CC) scenarios in Gus Creek, Pothole Creek, Rock Creek, and Upper Mainstem (UM) for the entire study period. Individual influences from stream temperature and flow were considered for single scenarios of FH and CC. Scenarios include manipulations of stream temperature and flow regimes (see Methods for details). Values of summer biomass by year were averaged for five replicate simulations and were analyzed using Wilcoxon signed rank test (V) with continuity correction resulting in a pseudomedian of difference between group 1 and group 2 (Δ). Significant p-values in bold (alpha ≤ 0.05). (DOCX) [file pone.0135334.s006.docx]

**S3 Table. Pairwise Comparisons of Summer Biomass of Trout.**

|  |  |  |  | entire 63 years | | | |
| --- | --- | --- | --- | --- | --- | --- | --- |
| stream | group 1 | group 2 | 95% CI | ∆ (g) | V | p-value |  |
| Gus | baseline | FH (flow + temperature) | -782, -315 | -542 | 370 | **<0.001** |  |
|  | FH (only flow) | FH (only temperature) | 87, 473 | 272 | 1417 | **0.005** |  |
|  | FH (only flow) | FH (flow + temperature) | -354, 104 | -121 | 848 | 0.28 |  |
|  | FH (only temperature) | FH (flow + temperature) | -620, -151 | -390 | 554 | **0.002** |  |
|  | baseline | CC (flow + temperature) | -578, -22 | -285 | 688 | **0.03** |  |
|  | CC (only flow) | CC (only temperature) | -1147, -587 | -869 | 283 | **<0.001** |  |
|  | CC (only flow) | CC (flow + temperature) | -855, -245 | -539 | 507 | **0.001** |  |
|  | CC (only temperature) | CC (flow + temperature) | 62, 709 | 421 | 1355 | **0.02** |  |
|  | baseline | FH + CC | -1125, 587 | -852 | 213 | **<0.001** |  |
| Pothole | baseline | FH (flow + temperature) | -103, -8 | -51 | 673 | **0.02** |  |
|  | FH (only flow) | FH (only temperature) | 44, 121 | 81 | 1619 | **<0.001** |  |
|  | FH (only flow) | FH (flow + temperature) | 254, 370 | 310 | 1996 | **<0.001** |  |
|  | FH (only temperature) | FH (flow + temperature) | 178, 285 | 231 | 1899 | **<0.001** |  |
|  | baseline | CC (flow + temperature) | 70, 176 | 125 | 1616 | **<0.001** |  |
|  | CC (only flow) | CC (only temperature) | -66, 34 | -18 | 913 | 0.52 |  |
|  | CC (only flow) | CC (flow + temperature) | 265, 370 | 314 | 2004 | **<0.001** |  |
|  | CC (only temperature) | CC (flow + temperature) | 277, 369 | 320 | 2006 | **<0.001** |  |
|  | baseline | FH + CC | 14, 113 | 63 | 1366 | **0.01** |  |
| Rock | baseline | FH (flow + temperature) | -54, 95 | 25 | 1124 | 0.43 |  |
|  | FH (only flow) | FH (only temperature) | 35, 129 | 82 | 1468 | **0.002** |  |
|  | FH (only flow) | FH (flow + temperature) | 45, 192 | 113 | 1458 | **0.002** |  |
|  | FH (only temperature) | FH (flow + temperature) | -21, 110 | 44 | 1216 | 0.16 |  |
|  | baseline | CC (flow + temperature) | 48, 190 | 119 | 1473 | **0.001** |  |
|  | CC (only flow) | CC (only temperature) | -133, 2 | -61 | 739 | 0.07 |  |
|  | CC (only flow) | CC (flow + temperature) | -33, 85 | 24 | 1136 | 0.38 |  |
|  | CC (only temperature) | CC (flow + temperature) | 41, 138 | 88 | 1532 | **<0.001** |  |
|  | baseline | FH + CC | -40, 143 | 50 | 1153 | 0.32 |  |
| UM | baseline | FH (flow + temperature) | -98, 165 | 15 | 1046 | 0.80 |  |
|  | FH (only flow) | FH (only temperature) | 807, 1086 | 941 | 2001 | **<0.001** |  |
|  | FH (only flow) | FH (flow + temperature) | -213, -43 | -130 | 628 | **0.01** |  |
|  | FH (only temperature) | FH (flow + temperature) | -1188, -934 | -1056 | 0 | **<0.001** |  |
|  | baseline | CC (flow + temperature) | 16, 395 | 213 | 1318 | **0.03** |  |
|  | CC (only flow) | CC (only temperature) | -123, 299 | 84 | 1109 | 0.49 |  |
|  | CC (only flow) | CC (flow + temperature) | -188, 149 | -29 | 957 | 0.73 |  |
|  | CC (only temperature) | CC (flow + temperature) | -201, -32 | -108 | 605 | **0.006** |  |
|  | baseline | FH + CC | 41, 658 | 341 | 1356 | **0.017** |  |

Pairwise comparisons of total summer biomass (g) of trout for baseline, forest harvest (FH), climate change (CC), and combined (FH + CC) scenarios in Gus Creek, Pothole Creek, Rock Creek, and Upper Mainstem (UM) for the entire study period. Individual influences from stream temperature and flow were considered for single scenarios of FH and CC. Scenarios include manipulations of stream temperature and flow regimes (see Methods for details). Values of summer biomass by year were averaged for five replicate simulations and were analyzed using Wilcoxon signed rank test (V) with continuity correction resulting in a pseudomedian of difference between group 1 and group 2 (∆). Significant p-values in bold (alpha ≤ 0.05).
