# Supplementary material for: Local Variability Mediates Vulnerability of Trout Populations to Land Use and Climate Change
Source: PLoS One. 2015 Aug 21;10(8):e0135334. doi: 10.1371/journal.pone.0135334 (PMC4546676; doi:10.1371/journal.pone.0135334)
Supplement: S4 Table — Median DOY of fry emergence of trout for forest harvest (FH), climate change (CC), and combined (FH + CC) scenarios in four modeled streams, including Gus Creek, Pothole Creek, Rock Creek, and Upper Mainstem (UM). Annual trends in fry emergence (days/decade) were averages of the five replicate simulations and were analyzed using the Mann-Kendall test and p-values were corrected for serial correlation for dates of the 1st harvest, 2nd harvest, and the entire study period. Scenarios include manipulations of stream temperature and flow regimes (see Methods for details). Significant p-values in bold (alpha ≤ 0.05) represent increasing or decreasing trends. Magnitude is the Sen slope (days/decade) over time. (DOCX) [file pone.0135334.s007.docx]

**S4 Table. Trends in Fry Emergence of Trout across Scenarios.**

|  |  | 1^st^ harvest | | | 2^nd^ harvest | | | entire 63 years | | |
| --- | --- | --- | --- | --- | --- | --- | --- | --- | --- | --- |
| stream | scenario | tau | p-value | slope | tau | p-value | slope | tau | P-value | slope |
| Gus | FH | 0.06 | 0.52 | 1 | 0.08 | 0.67 | 2 | 0.00 | 0.97 | 0 |
|  | CC | -0.38 | **0.02** | -12 | -0.29 | 0.07 | -3 | -0.68 | **<0.001** | -6 |
|  | FH+CC | -0.47 | **0.01** | -9 | -0.22 | 0.21 | -3 | -0.67 | **<0.001** | -5 |
| Pothole | FH | 0.22 | **0.02** | 3 | 0.00 | 0.97 | 1 | -0.15 | 0.07 | -1 |
|  | CC | -0.39 | **0.03** | -7 | -0.37 | **0.03** | -4 | -0.68 | **<0.001** | -5 |
|  | FH+CC | -0.17 | 0.06 | -3 | -0.42 | **0.01** | -4 | -0.63 | **<0.001** | -5 |
| Rock | FH | 0.24 | 0.14 | 3 | 0.10 | 0.58 | 2 | 0.04 | 0.70 | 0 |
|  | CC | -0.41 | **<0.001** | -7 | -0.24 | 0.16 | -5 | -0.61 | **<0.001** | -4 |
|  | FH+CC | -0.34 | 0.10 | -6 | -0.12 | 0.50 | -2 | -0.59 | **<0.001** | -4 |
| UM | FH | 0.04 | 0.83 | 0 | 0.05 | 0.88 | -3 | 0.10 | 0.39 | 0 |
|  | CC | -0.26 | 0.12 | -5 | -0.24 | 0.35 | -18 | -0.50 | **<0.001** | -7 |
|  | FH+CC | -0.19 | 0.26 | -3 | -0.24 | 0.35 | -26 | -0.33 | **<0.001** | -4 |
|  |  |  |  |  |  |  |  |  |  |  |

Median DOY of fry emergence of trout for forest harvest (FH), climate change (CC), and combined (FH + CC) scenarios in four modeled streams, including Gus Creek, Pothole Creek, Rock Creek, and Upper Mainstem (UM). Annual trends in fry emergence (days/decade) were averages of the five replicate simulations and were analyzed using the Mann-Kendall test and p-values were corrected for serial correlation for dates of the 1^st^ harvest, 2^nd^ harvest, and the entire study period. Scenarios include manipulations of stream temperature and flow regimes (see Methods for details). Significant p-values in bold (alpha ≤ 0.05) represent increasing or decreasing trends. Magnitude is the Sen slope (days/decade) over time.
