# Supplementary material for: Local Variability Mediates Vulnerability of Trout Populations to Land Use and Climate Change
Source: PLoS One. 2015 Aug 21;10(8):e0135334. doi: 10.1371/journal.pone.0135334 (PMC4546676; doi:10.1371/journal.pone.0135334)
Supplement: S5 Table — Pairwise comparisons of winter survival (proportion of total in that age class that survive), growth (cm/month), and biomass (g) for three age classes (ages 1, 2, 3+) of trout in relation to baseline for forest harvest (FH), climate change (CC), and combined (FH + CC) scenarios in four modeled streams over 63 years. Streams include Gus Creek, Pothole Creek, Rock Creek, and Upper Mainstem (UM). Scenarios include manipulations of stream temperature and flow regimes (see methods for detail). The magnitude of change for each scenario relative to baseline is an average of five replicate simulations and is calculated as: [(median scenario − median baseline)/median baseline]*100. Winter is January and February. Age classes during winter were restricted to ages 1+ because the model assigns an additional year to each trout on January 1st every year, thus there are no age 0 trout in winter. Responses were analyzed using Kruskal-Wallis one-way analysis of variance on ranks. Negative values indicate that the values for the response of the scenario of interest is lower than baseline values for that stream and positive values indicate that it is higher than baseline. Only significant responses are shown (alpha ≤ 0.05). (DOCX) [file pone.0135334.s008.docx]

**S5 Table. Pairwise Comparisons of Winter Survival, Growth, and Biomass by Trout Age Class.**

|  |  | age 1 | | | age 2 | | | age 3+ | | |
| --- | --- | --- | --- | --- | --- | --- | --- | --- | --- | --- |
| parameter | stream | FH | CC | FH + CC | FH | CC | FH+ CC | FH | CC | FH + CC |
| survival | Gus | - | 1% | - | - | - | - | - | - | - |
|  | Pothole | - | - | - | - | - | - | - | - | - |
|  | Rock | - | 3% | - | - | - | - | - | - | - |
|  | UM | -1% | - | - | - | - | - | - | - | - |
|  |  |  |  |  |  |  |  |  |  |  |
| growth | Gus | - | 10% | 8% | - | -14% | -15% | - | - | - |
|  | Pothole | -1% | -5% | -6% | - | - | - | - | - | - |
|  | Rock | -11% | - | 4% | -11% | -29% | -30% | -31% | -100% | -100% |
|  | UM | -2% | -25% | -20% | - | -22% | -44% | - | - | - |
|  |  |  |  |  |  |  |  |  |  |  |
| biomass | Gus | - | 26% | 37% | - | - | 8% | 8% | 17% | -8% |
|  | Pothole | - | 3% | 3% | 4% | -22% | -18% | - | -22% | - |
|  | Rock | -4% | 17% | 19% | - | -49% | - | - | -74% | -48% |
|  | UM | - | - | - | - | -34% | -38% | -34% | - | -34% |

Pairwise comparisons of winter survival (proportion of total in that age class that survive), growth (cm/month), and biomass (g) for three age classes (ages 1, 2, 3+) of trout in relation to baseline for forest harvest (FH), climate change (CC), and combined (FH + CC) scenarios in four modeled streams over 63 years. Streams include Gus Creek, Pothole Creek, Rock Creek, and Upper Mainstem (UM). Scenarios include manipulations of stream temperature and flow regimes (see methods for detail). The magnitude of change for each scenario relative to baseline is an average of five replicate simulations and is calculated as: [(median scenario - median baseline)/median baseline]*100. Winter is January and February. Age classes during winter were restricted to ages 1+ because the model assigns an additional year to each trout on January 1^st^ every year, thus there are no age 0 trout in winter. Responses were analyzed using Kruskal-Wallis one-way analysis of variance on ranks. Negative values indicate that the values for the response of the scenario of interest is lower than baseline values for that stream and positive values indicate that it is higher than baseline. Only significant responses are shown (alpha ≤ 0.05).
